# Incorporating Functional Genomic Information in Genetic Association Studies Using an Empirical Bayes Approach

**DOI:** 10.1002/gepi.21956

**Published:** 2016-02-01

**Authors:** Amy V. Spencer, Angela Cox, Wei‐Yu Lin, Douglas F. Easton, Kyriaki Michailidou, Kevin Walters

**Affiliations:** ^1^ Advanced Analytics CentreGlobal Medicines DevelopmentAstraZenecaAlderley ParkMacclesfieldUnited Kingdom; ^2^ School of Mathematics and StatisticsUniversity of SheffieldSheffieldUnited Kingdom; ^3^ Department of OncologySheffield Cancer Research CentreUniversity of Sheffield Medical SchoolBeech Hill RoadSheffieldUnited Kingdom; ^4^ Cardiovascular Epidemiology UnitDepartment of Public Health and Primary CareUniversity of CambridgeCambridgeUnited Kingdom; ^5^ Department of Public Health and Primary CareCentre for Cancer Genetic EpidemiologyUniversity of CambridgeCambridgeUnited Kingdom; ^6^ Department of OncologyCentre for Cancer Genetic EpidemiologyUniversity of CambridgeCambridgeUnited Kingdom

**Keywords:** fine mapping, Bayes factors, genetic association, functional information, empirical Bayes

## Abstract

There is a large amount of functional genetic data available, which can be used to inform fine‐mapping association studies (in diseases with well‐characterised disease pathways). Single nucleotide polymorphism (SNP) prioritization via Bayes factors is attractive because prior information can inform the effect size or the prior probability of causal association. This approach requires the specification of the effect size. If the information needed to estimate a priori the probability density for the effect sizes for causal SNPs in a genomic region isn't consistent or isn't available, then specifying a prior variance for the effect sizes is challenging. We propose both an empirical method to estimate this prior variance, and a coherent approach to using SNP‐level functional data, to inform the prior probability of causal association. Through simulation we show that when ranking SNPs by our empirical Bayes factor in a fine‐mapping study, the causal SNP rank is generally as high or higher than the rank using Bayes factors with other plausible values of the prior variance. Importantly, we also show that assigning SNP‐specific prior probabilities of association based on expert prior functional knowledge of the disease mechanism can lead to improved causal SNPs ranks compared to ranking with identical prior probabilities of association. We demonstrate the use of our methods by applying the methods to the fine mapping of the CASP8 region of chromosome 2 using genotype data from the Collaborative Oncological Gene‐Environment Study (COGS) Consortium. The data we analysed included approximately 46,000 breast cancer case and 43,000 healthy control samples.

## Introduction

Association between genetic variants and the occurrence of a disease is typically analysed using *P*‐values from purely likelihood‐based hypothesis tests such as Wald or Cochran‐Armitage tests. These can be used to rank variants in order of association in both genome‐wide association studies, and fine‐mapping studies focussing on a much smaller genetic region, in which causal association with the disease has already been convincingly established. Fine‐mapping studies that fail to take into account known functional information are arguably not making full use of all the available data.

Bayesian analysis provides a convenient framework for combining prior information from multiple sources, and Bayes factors (BFs) [Kass and Raftery, 1995] are now routinely used to identify genetic variants in genetic association studies [Stephens and Balding, 2009; Maller et al., 2012; Spencer et al., 2015], but there are several practical difficulties. In particular, the marginal likelihoods required to calculate the BF often lead to intractable integrals, meaning that the BF must be approximated. With large genetic studies, this is not problematic because asymptotic approximations exist. Two approximations, the Laplace approximation and the Wakefield BF (WBF) approximation, are currently available. The Laplace approximation is integrated in the software snptest2 [Marchini et al., 2007], and the WBF approximation [Wakefield, 2008, 2009] has a simple algebraic form. To calculate the BF, the prior distribution of the log odds ratio (logOR) for effect size must be specified, and the results can be highly variable dependent on this prior. The prior is usually taken to be Gaussian with a known variance. Specifying this variance may be difficult for alleles involved in common diseases. One way to deal with this uncertainty is to add further layers to the prior hierarchy by letting the prior variance take a probability distribution [Spencer et al., 2015]. Here, we demonstrate an empirical Bayes method that can be used as an alternative if the prior variance is not well characterised, and which can produce higher causal single nucleotide polymorphism (SNP) ranks than using many values of the prior variance within a realistic range.

We consider the suitability of posterior probabilities of association as a statistic for ranking genetic variants. To calculate these posterior probabilities of association, prior odds are updated through the BF. There is now a vast array of functional information available on the genome, for example, that from the encode project [Encode Project Consortium, 2011], which may be used to inform these probabilities. How to translate such information into prior probabilities is not clear. In this paper, we attempt to address the practical issues of choosing both the variance of the prior distribution of the logOR and the prior probabilities of association.

## Materials and Methods

Following a fine‐mapping association study, we wish to rank variants in order of $\Delta_{i}$, the posterior probability that variant *i* is causally associated with the disease. Bayes theorem allows us to express the posterior odds of association as $\Delta_{i}/\left(1-\Delta_{i}\right)=\delta_{i}/\left(1-\delta_{i}\right)\times \mathrm{BF}$ [Kass and Raftery, 1995], where $\delta_{i}$ is the prior probability of causal association for variant *i* and the BF is the ratio of the marginal likelihoods under two differing hypotheses:

(1) $$\mathrm{BF}=\frac{f(\mathrm{data}|H_{1})}{f(\mathrm{data}|H_{0})}.$$

In univariate logistic regression models we assume that, for variant *i*, the probability ($y_{il}$) of subject *l* with $x_{il}$ copies of the minor allele being a case is $y_{il}=e^{\beta_{0i}+\beta_{1i}x_{il}}/\left(1+e^{\beta_{0i}+\beta_{1i}x_{il}}\right)$. The usual test is then $H_{0}:\beta_{1i}=0$ and $H_{1}:\beta_{1i}\neq 0$ [Stephens and Balding, 2009], where $\beta_{1i}$ is the natural logarithm of the odds ratio of the variant *i*. In order to calculate $\Delta_{i}$ for variant *i*, a prior distribution on the logOR, $\beta_{1i}$, is needed, and a prior probability of causal association $\delta_{i}$ has to be specified.

### Constructing Empirical BF

We focus on using the WBF approximation, which assumes a prior of the form $\beta_{1}∼N\left(0,W\right)$. Note that for the sake of brevity, we drop the *i* subscript on $\beta_{1i}$, where the meaning is clear. Asymptotically, the estimate of the logOR is distributed $\hat{\beta_{1}}∼N\left(\beta_{1},V\right)$, and Wakefield showed that the BF can be written as

(2) $$\mathrm{WBF}=\sqrt{\frac{V}{V+W}}\exp\left(\frac{{\hat{\beta_{1}}}^{2}W}{2V(V+W)}\right).$$

To aid our calculations, this is the reciprocal of the approximation given by Wakefield in his papers [Wakefield, 2008, 2009].

Although this approximation is convenient, it still requires a value for *W*, the prior variance of the logOR, for each variant to be specified a priori, which may be difficult. Spencer et al. [2015] showed the ranking of SNPs is potentially very sensitive to the choice of *W*. This sensitivity is a potential drawback in studies utilising BFs where there is functional information to inform prior probabilities of association but where there are few previous studies available to use to estimate likely effect sizes a priori.

A standard Bayesian method would require the prior distribution (and therefore *W*) to be chosen before the data are obtained. Empirical Bayes is an alternative approach that involves estimating prior hyper‐parameters (in this case *W*) from the data. In the case where a suitable value for *W* is difficult to choose, we propose choosing *W* to maximise the marginal likelihood f( data |W). The drawback of using an empirical Bayes approach is that the data are effectively used twice: to inform the prior and also in the likelihood. We emphasize that where reliable information is available to inform the prior effect size this should be used instead. In the Appendix we show that f( data |W)∝ WBF  when considered as a function of *W*. In the Appendix we also show that WBF, when considered as a function of *W*, is maximised when

(3) WEB=β1^2−V if β1^2≥V0 otherwise .

We use the notation $W_{\mathrm{EB}}$ for this empirical Bayes inspired value of *W* and ${\mathrm{BF}}_{\mathrm{EB}}$ for the associated BF. If $z^{2}={\hat{\beta_{1}}}^{2}/V$, it follows that

(4) BFEB=1z exp z2−12 if β1^2≥V1 otherwise .

This is not a standard empirical Bayes approach because we are using ${\hat{\beta_{1}}}^{2}$ and *V* as surrogates for the data at each SNP. These values vary by SNP and there are many ways of selecting the values to use for all SNPs. An obvious approach is to use the values for the causal SNP. To do this one would need to calculate $W_{\mathrm{EB}}$ using ${\hat{\beta_{1}}}_{c}$ and $V_{c}$, the $\hat{\beta_{1}}$ and *V* values specific to the causal variant, which are of course unknown. We suggest several empirical surrogates for these values, and give the results of an investigation comparing them in the Results section. For the estimate of $V_{c}$, we suggest using the median *V* estimated for all variants in the dataset and denote it as $V_{m}$.

To approximate ${\hat{\beta_{1}}}_{c}$, we use the fact that the causal variant is likely to be in higher linkage disequilibrium (LD) with variants that are highly associated with the phenotype of interest than other variants in the region. We use the likelihoods of the single‐variant logistic regression models as a simple measure of association. One option is to use $\hat{\beta_{1}}$ of the variant that produces the model with the largest likelihood (${\hat{\beta_{1}}}_{max}$). However, we have previously shown that ranking genetic variants in a fine‐mapping scenario based on LD with this variant is not efficacious [Spencer et al., 2014]. Therefore, we also considered estimating ${\hat{\beta_{1}}}_{c}$ by taking the top *p*% of variants ranked by likelihood and using the median value of $|\hat{\beta_{1}}|$ for this group, denoted ${\hat{\beta_{1}}}_{p}$. The choice of the median was due to the lower bound of zero and the skewed distribution of ${\hat{\beta_{1}}}_{p}$. In order to compare the effectiveness of the different approximations for ${\hat{\beta_{1}}}_{c}$, we analysed simulated data using WBFs calculated with $W_{\mathrm{EB}}$ as the ranking statistic. The same simulated data were analysed using the various suggested approximations, including different values for *p*.

### Data Used in the Study

We simulated case‐control genotypes, where the ‘true’ causal variant is known, using hapgen2 [Spencer et al., 2009; Su et al., 2011] with reference haplotypes from the 1000 Genomes Study [Altshuler et al., 2010]. Specifically we simulated 2,871 SNPs in a one mega‐base region (from 201,566,128 to 202,566,128 bases in the Hg19 build) around the *CASP8* region on chromosome 2. We considered six minor allele frequency (MAF)/odds ratio (OR) combinations for the causal SNP: an MAF of 0.08 and 0.18 and a per‐allele OR of 1.06, 1.1 and 1.14. For the MAF of 0.08 we simulated 10,000 cases and 10,000 controls. When the simulated causal SNP had an MAF of 0.08 and an odds ratio of 1.10 the median value of *V* was 0.00208 and was very similar for the other two odds ratio for this MAF. For the MAF of 0.18 we simulated 5,000 cases and 5,000 controls. When the simulated causal SNP had an MAF of 0.18 and an odds ratio of 1.10 the median value of *V* was 0.00143 and was very similar for the other two odds ratio for this MAF. We assumed a multiplicative genetic model throughout.

We define filtering to be the ranking of all genetic variants by a statistic such as Δ and removal of all variants below a threshold [Spencer et al., 2014]. To assess our methods, we carried out variant filtering on the simulated datasets. We plot true‐positive rates (TPR) for each false‐positive rate (FPR) using receiver operating characteristic (ROC) curves in 1,000 simulated fine‐mapped datasets and compare this to the ROC generated by using other methods to compare ranking efficacy. We combine the data from each set of 1,000 simulated datasets into a single ROC curve using a method called ‘threshold averaging’ [Fawcett, 2006]. We present the mean TPR for each FPR considered.

In a recent study by the Collaborative Oncological Gene‐Environment Study (COGS) Consortium, several regions of the genome were fine mapped using the iCOGS array (a specially developed Illumina array), and analysed for association with breast cancer [Michailidou et al., 2013]. We utilised the data from the *CASP8* region and, after quality control checks, we had genotyped information for 501 variants and imputed genotype probabilities for a further 1,232 variants between base positions 201,500,074 and 202,569,992 (imputation carried out using impute2 [Marchini and Howie, 2010]). The genotypes of these 1,733 variants were available for a total sample size of 89,050 subjects (46,450 cases and 42,600 controls).

### Functional Data Used to Inform Prior Odds of Association

Our aim is to rank variants by Δ values and in order to calculate these we also need to assign a δ value to each variant. If nothing was previously known about the genotyped variants they could all be assigned the same δ and the ranking by Δ would be equivalent to ranking by BF (using association information from the data alone). However, much investigation has been done into the functionality of genetic variants and there is a large amount of information publicly available. One of the richest sources of such information is the Encyclopaedia of DNA Elements (encode) [Encode Project Consortium, 2011], which is available to view using the UCSC Genome Browser. It contains data for a huge number of variables recorded at the SNP level, some of which are likely to be related to whether or not an SNP will have a deleterious effect on a disease. There are many ways that δ values may be generated from the data in encode, but we outline one general method here.

The δ values we use are expert‐specific subjective probabilities. An expert here meaning a geneticist with expertise in the particular disease area. Our method employs elicitation, which involves working with the expert to formulate a numeric representation of their beliefs, in this case about δ values for all the genetic variants. Alternatively, multiple experts may be used, but this adds to the complexity by necessitating the combination of multiple opinions into a single prior. This also slightly changes the problem, as a combination prior is not a subjective prior in the same way that a single expert's opinion is, and therefore the resulting value or distribution does not have an intuitive meaning. This problem is discussed in a thorough review on the topic of elicitation by Garthwaite et al. [2005].

### Using Functional Information to Inform Prior Odds

Elicitation can be used to specify a fixed value or a probability distribution. It is unrealistic to elicit δ values for individual SNPs so we propose grouping SNPs into groups of similar broad functionality. Therefore we need to elicit only a few δ values, one for each SNP group. We do this using the following procedure:

*Step 1*: The expert should choose a subset of the encode variables relevant to the disease of interest.

*Step 2*: If appropriate, group the encode variables into summary variables indicating broader functionality and formulate binary decision rules based on the values of the summary variables to partition SNPs into those ‘more likely’ and ‘less likely’ to be causal.

*Step 3*: Construct a tree with the summary variables (in an appropriate order) as the nodes and the binary decision rules as the branches. Use the tree to partition the SNPs into a small number (*J*) of prior probability groups, ordered from ‘very unlikely’ to ‘very likely’ to be causal. See Figure 2 for an example of one we constructed for the SNPs in the CASP8 region. Let $\delta_{\left[j\right]}$ be the prior probability of a group *j* SNP being causal.

*Step 4*: Elicit from the expert the prior probability (*p*_0_) that none of the variants analysed is causal.

Define *N* to be the total number of SNPs to partition into the *J* groups and $n_{j}$ to be the number of SNPs in group *j*. We assume that the event that SNP *i* is causal, is independent of the event that SNP *k* is causal (i≠k). If we further assume that $\delta_{\left[1\right]},...,\delta_{\left[J\right]}$ are small then we have

$$\begin{array}{ccc} p_{0} & = & \prod_{i=1}^{n_{1}}\left(1-\delta_{\left[1\right]}\right)\prod_{i=1}^{n_{2}}\left(1-\delta_{\left[2\right]}\right)...\prod_{i=1}^{n_{J}}\left(1-\delta_{\left[J\right]}\right)\\ & = & \prod_{j=1}^{J}{\left(1-\delta_{\left[j\right]}\right)}^{n_{j}}\approx 1-\sum_{j=1}^{J}n_{j}\delta_{\left[j\right]}+O\left(\delta^{2}\right). \end{array}$$

To calculate the values of $\delta_{\left[1\right]},...,\delta_{\left[J\right]}$ we have to solve $1-p_{0}=\sum_{j=1}^{J}n_{j}\delta_{\left[j\right]}$. If we make the further assumption that $\delta_{\left[j+1\right]}=R\delta_{\left[j\right]}$ for j=1,...,J−1 then we can get

(5) $$\delta_{\left[1\right]}=\frac{1-p_{0}}{n_{J}R^{J-1}+n_{J-1}R^{J-2}+\cdots +n_{2}R+n_{1}}$$

and the remaining $\delta_{\left[j\right]}$ values are then calculated using $\delta_{\left[j+1\right]}=R\delta_{\left[j\right]}$. The accuracy of Equation (5) depends on the relative sizes of $n_{j}$ and $\delta_{\left[j\right]}$. In the binomial expansion ${\left(1-\delta_{\left[j\right]}\right)}^{n_{j}}$ the ratio of the (u+1) th  to the *u*th term is $\delta_{\left[j\right]}\left(n_{j}-u\right)/\left(u+1\right)$ so the smaller the $\delta_{\left[j\right]}n_{j}=R^{j-1}\delta_{\left[1\right]}n_{j}$ is, the better Equation (5) is as an approximation. The value of *j* for which $R^{j-1}/n_{j}$ is largest also provides an indication of where extra terms may be needed in the binomial expansions. The resulting values derived from Equation (5) should be checked to ensure that $p_{0}\approx \prod_{j=1}^{J}{\left(1-\delta_{\left[j\right]}\right)}^{n_{j}}$. If the approximation appears to be poor, then quadratic terms can be considered in the binomial expansion yielding a quadratic equation $a\delta_{\left[1\right]}^{2}+b\delta_{\left[1\right]}+c=0$, where a=∑j=1Jnj2R2j−2+∑j=1J−1∑j*>jJnjnj*Rj+j*−2, $b=-\sum_{j=1}^{J}R^{j-1}n_{j}$ and $c=1-p_{0}$. A poor second‐order approximation could yield a quadratic without real roots. In this case a numerical method such as the uniroot function in R should be used to solve $p_{0}=\prod_{j=1}^{J}{\left(1-\delta_{\left[j\right]}\right)}^{n_{j}}$, where $\delta_{\left[j+1\right]}=R\delta_{\left[j\right]}$.

Assuming that the events that SNP *i* is causal and SNP *k* is causal are independent takes no account of the likely number of causal SNPs in the region. If it is desirable to incorporate prior knowledge about the number of causal SNPs, then this can be achieved with some modification to Equation (5). We present a method in which up to two causal SNPs are present in the genomic region. Let $p_{m}$ represent the prior probability that there are *m* causal SNPs in the region. Then it follows that

(6) 1=p0+p1∑j=1Jnjδ[j]+p2∑j=1Jnj2δ[j]2+∑j=1J−1∑k=j+1Jnjnkδjδk.

We can also relax the somewhat rigid assumption that $\delta_{\left[j+1\right]}=R\delta_{\left[j\right]}$ and instead allow group‐specific multiplicative increases by letting $\delta_{\left[j+1\right]}=R_{j}\delta_{\left[j\right]}$. It then follows that

(7) 1=p0+p1n1+∑j=2Jnj∏k=1j−1Rkδ[1]+p2n12+∑j=2Jnj2∏k=1j−1Rk2+∑k=2Jn1nk∏j*=1k−1Rj*+∑j=2J−1∑k=j+1Jnjnk∏j*=1j−1Rj*2∏j*=jkRj*δ[1]2.

This yields a quadratic in $\delta_{\left[1\right]}^{2}$. We used Equation (5) with our expert geneticist to assign probabilities to the *CASP8* region variants because we didn't have reliable information about the number of causal SNPs in the region. In addition the expert had no reason to believe that the multiplicative increase in prior probabilities was unrealistic.

After carrying out the elicitation and assigning the SNPs to J=4 groups, we selected one SNP from each group with similar MAF (between 0.037 and 0.049). The selected SNPs from each of the four groups were chosen to be in very high LD with each other. All pairwise $D^{\prime }$ values for these four causal SNPs were 1 except for one pair with $D^{\prime }=0.916$ ($r^{2}=0.839$). We simulated sets of 1,000 datasets with the selected SNP in each group as the causal SNP with a per‐allele OR of 1.1 and then analysed the data using WBF, with a prior on the logOR of $N(0,W_{\mathrm{EB}}={\hat{\beta_{1}}}_{p=30}^{2}-V_{m})$. After calculating Δ values for all SNPs, we ranked and filtered them using these values. By choosing SNPs in very high LD, we limit the effect of the underlying LD structure when using different causal variants so that the differences seen are the result of the different δ values assigned. This was also checked by carrying out BF filtering on the four sets of simulations.

## Results

### SNP Filtering in the Simulated Data Using Empirical BFs

**Figure 1:**
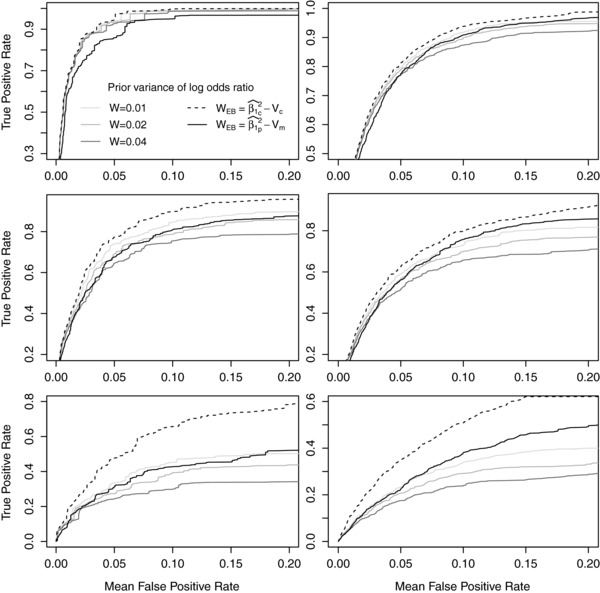
Receiver operating characteristic (ROC) curves of BF filtering results, each using the Wakefield approximation and N(0,W) prior for the log odds ratio with a different value of *W*, some based on empirical information ($W_{\mathrm{EB}}$). Those that use empirical Bayes methods have subscripts denoting whether they are based on the causal SNP (c) or the median across all SNPs (m) or the median across the top p% of SNPs (p), where in this case p=30. The filtering was carried out on 1,000 datasets simulated using the LD structure of the *CASP8* region for six scenarios. In the left (right) column the total sample size was 10,000 (20,000) and the causal SNP had an MAF of 0.18 (0.08). In the top, middle and bottom rows, the causal SNP had a per‐allele OR of 1.14, 1.10 and 1.06, respectively.

Figure 1 shows the results of filtering for the six simulated scenarios (MAF=0.08, 0.18 and OR=1.06, 1.10, 1.14) using the WBF where the prior variance of the logOR (*W*) is equal for all SNPs in a dataset. The solid grey ROC curves show the results for W=0.01,0.02 and 0.04. The dashed line shows the results of using $W_{\mathrm{EB}}={\hat{\beta_{1}}}_{c}^{2}-V_{c}$, where ${\hat{\beta_{1}}}_{c}$ and $V_{c}$ relates to the unknown values for the causal SNP. The solid black line shows the results of using $W_{\mathrm{EB}}={\hat{\beta_{1}}}_{p}^{2}-V_{m}$, where $V_{m}$ is the median of *V* across all SNPs and ${\hat{\beta_{1}}}_{p}$ is the median of the $|\hat{\beta_{1}}|$ values of the top p% of SNPs ranked by likelihood (here we use p=30). $W_{\mathrm{EB}}={\hat{\beta_{1}}}_{c}^{2}-V_{c}$ represents the theoretical upper bound corresponding to the case where ${\hat{\beta_{1}}}_{c}$ and $V_{c}$ are known. In Figure 1 we focus on FPR ⩽ 0.2 as this represents the most interesting parts of the ROC space in fine mapping. The results do not qualitatively change at higher FPRs. Table 1 shows the distribution of the $W_{\mathrm{EB}}$ values across the 1,000 simulated datasets for each of the six MAF/OR scenarios. The minimum value of $W_{\mathrm{EB}}$ is zero in each of the six scenarios.

**Table 1:** Percentiles of the distribution, across simulated datasets, of $W_{\mathrm{EB}}$ for each of the six different odds ratio, MAF and sample size scenarios considered

		Simulated odds ratio		
Sample size (SS) and MAF	Percentile of $W_{\mathrm{EB}}$	1.06	1.10	1.14
SS = 20,000	25	0.0021	0.0025	0.0035
MAF = 0.08	50	0.0036	0.0043	0.0056
	75	0.0057	0.0061	0.0079
	100	0.0210	0.0172	0.0243
SS = 10,000	25	0.0026	0.0023	0.0023
MAF = 0.18	50	0.0062	0.0050	0.0050
	75	0.0111	0.0085	0.0084
	100	0.0355	0.0453	0.0270

Using $W_{\mathrm{EB}}={\hat{\beta_{1}}}_{30}^{2}-V_{m}$ generally performs well when the causal variant has an MAF of 0.08 (right‐hand column of Fig. 1) compared to other values of *W* considered. When the simulated causal SNP MAF is 0.18 (left‐hand column), the performance was competitive in two scenarios (lower effect sizes) but poor when the causal SNP OR was 1.14. The reasons for the relatively poor performance in this one case are not clear. We investigated different values of the percentile *p* in $W_{\mathrm{EB}}$ and found values around p=30 generally gave the best performance in all six scenarios considered (results not shown).

**Figure 2:**
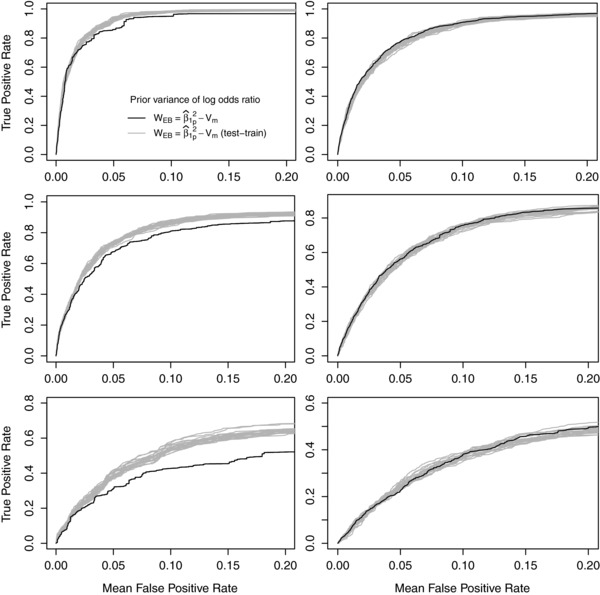
Receiver operating characteristic (ROC) curves of BF filtering results, each using the Wakefield approximation and N(0,W) prior for the log odds ratio with *W* based on empirical information ($W_{\mathrm{EB}}$). The subscripts denote whether they are based on the causal SNP (c) or the median across all SNPs (m) or the median across the top p% of SNPs (p), where in this case p=30. The filtering was carried out on 1,000 datasets simulated using the LD structure of the *CASP8* region for six scenarios. Twenty different partitions of the 1,000 datasets into 500 paired datasets were constructed. In each of these 20 partitions $W_{\mathrm{EB}}$ was calculated from one dataset of each pair and applied to the other dataset in the pair. The ROC curves for these 20 train‐test partitions are given by $W_{\mathrm{EB}}={\hat{\beta_{1}}}_{30}^{2}-V_{m}$ (test train). In the left (right) column the total sample size was 10,000 (20,000) and the causal SNP had an MAF of 0.18 (0.08). In the top, middle and bottom rows, the causal SNP had a per‐allele OR of 1.14, 1.10 and 1.06, respectively.

A potential criticism of our empirical Bayes approaches is that the same data get used twice, once to inform the prior via calculation of $W_{\mathrm{EB}}$ and then again in the calculation of the BF. This has the potential to lead to overfitting. To assess whether our use of empirical Bayes leads to overfitting we estimated $W_{\mathrm{EB}}$ on a training dataset and then used this value of $W_{\mathrm{EB}}$ in a test dataset of the same size. We randomly selected 500 pairs of training and test data from the 1,000 simulated datasets (for each MAF). Within each pair we estimated $W_{\mathrm{EB}}$ in the training data and used this value in the test dataset. We repeated this random sampling of 500 pairs 20 times and show the ROC curves for these 20 random samples in Figure 2. We consider the same six MAF/odds ratio scenarios presented in Figure 1. $W_{\mathrm{EB}}={\hat{\beta_{1}}}_{p}^{2}-V_{m}$ represents the case where $W_{\mathrm{EB}}$ is calculated and implemented on the same data and is reproduced from Figure 1 and $W_{\mathrm{EB}}={\hat{\beta_{1}}}_{p}^{2}-V_{m}$ (test train) represents our test‐train approach.

If our empirical Bayes approach leads to overfitting, we would observe a meaningful reduction in performance. There is no evidence of overfitting in the plots on the right of Figure 2, whilst there is some evidence of overfitting in the plots on the left of Figure 2, particularly when the odds ratio is 1.06. Our results suggest that the empirical Bayes approach can lead to overfitting when the causal SNP odds ratio is very small and the sample size is modest but should otherwise not suffer adversely from overfitting. It should be emphasised that we are not advocating this train‐test approach because it would require double the sample size compared to our standard empirical Bayes approach. We include it simply to determine the existence and extent of overfitting.

### SNP Filtering in the Simulated Data Using Posterior Probabilities

Using the steps outlined in the Methods section, we were able to assign prior probabilities of causal association to simulated SNPs in the *CASP8* region in the 1,000 genomes data. The encode variables that were chosen for *step 1* and the summary variables assigned in *step 2* are given in Table 2. There is a lot of missing data in the encode variables, with ‘gene’ being the only one we used that didn't have any values missing. The percentages missing for each variable are shown in Table 3. We dealt with missing values by replacing them with zeros (because all measurements were positive) under the assumption that they are missing because they didn't exceed the measurement threshold.

**Table 2:** Four summary variables to describe the SNPs in the 1 Mb region surrounding *CASP8*

Summary variable	Values of encode variables for which SNPs are likely to be causal	Values of encode variables for which SNPs are unlikely to be causal
Regional location	Gene given as *CASP8* or *ALS2CR12*	Elsewhere
Histone modification	H1: layeredGm12878H3k4me1StdSig ⩾5 or H2: layeredHmecH3k4me3StdSig $\geq e^{1.5}$ or H3: layeredHmecH3k27acStdSig $\geq e^{1.75}$	Otherwise
Availability	A1: TranscriptionGm12878 $\geq e^{1.5}$ or A2: TxnFactorChip ⩾100 or any indicator for A3: OpenChromSynthGm12878Pk	Otherwise
Conservation	Conservation value >0	Otherwise

These are determined based on the following variables downloaded from the encode database: gene, layeredGm12878H3k4me1StdSig, layeredHmecH3k4me3StdSig, layeredHmecH3k27acStdSig, TranscriptionGm12878, TxnFactorChip, OpenChromSynthGm12878Pk and Conservation. Values given in the table were used to determine how likely SNPs with that description/value are to be causal, compared with other SNPs in the region.

**Table 3:** Percentage missing data by ENCODE variable and summary variable used to partition the SNPs into groups

Summary variable	Regional location		Histone modification			Availability			Conservation
ENCODE variable	CASP8	ALS2CR12	H1	H2	H3	A1	A2	A3	Conservation
Percentage missing by variable	0	0	33.5	31.6	38.4	71.6	88.7	97.2	94.7
Percentage missing by summary variable	0		6.0			65.2			94.7

The ENCODE variables corresponding to H1–H3 and A1–A3 are specified in Table 2.

**Figure 3:**
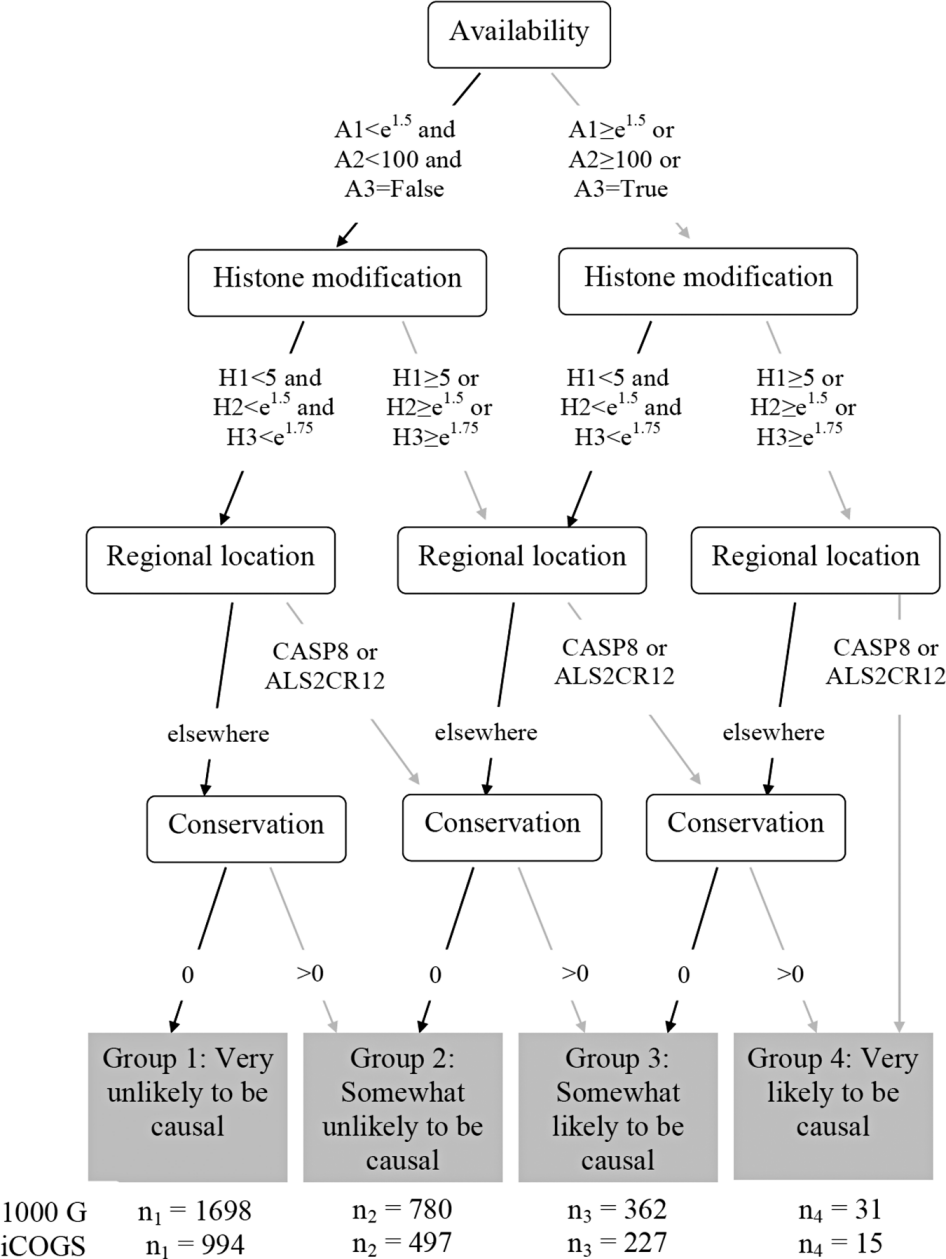
Flow diagram showing how SNPs in the *CASP8* region were divided into four groups, depending on four summary variables: *Regional location, Histone modification, Availability* and *Conservation*. The groups represent the subjective belief of a breast cancer geneticist about how likely SNPs are to be causal. The number of SNPs assigned to each group is given for both the simulated data based on the 1,000 genomes data, and for the iCOGS study in the last two lines.

We grouped our ENCODE variables into J=4 groups (*step 3*), depending on the SNP‐specific outcomes and the expert's decision rules relating to what thresholds to apply to each ENCODE variable within the summary variable (see Fig. 3). Of the N=2,871 SNPs, this resulted in $n_{1}=1,698$ SNPs being assigned to the ‘very unlikely to be causal’ group 1, $n_{2}=780$ to group 2, $n_{3}=362$ to group 3 and $n_{4}=31$ to the ‘very likely to be causal’ group 4.

Our expert specified the probability that there was no causal SNP in the region as approximately $p_{0}=0.4$ and specified the multiplicative increase in the prior probability of being causal in group j+1 compared to group *j* as R=5. Substituting these values into Equation (5) gives $\delta_{\left[1\right]}=3.2\times 10^{-5}$, from which we can infer using $\delta_{\left[j+1\right]}=R\delta_{\left[j\right]}$ that $\delta_{\left[2\right]}=1.6\times 10^{-4}$, $\delta_{\left[3\right]}=8\times 10^{-4}$ and $\delta_{\left[4\right]}=4\times 10^{-3}$.

**Figure 4:**
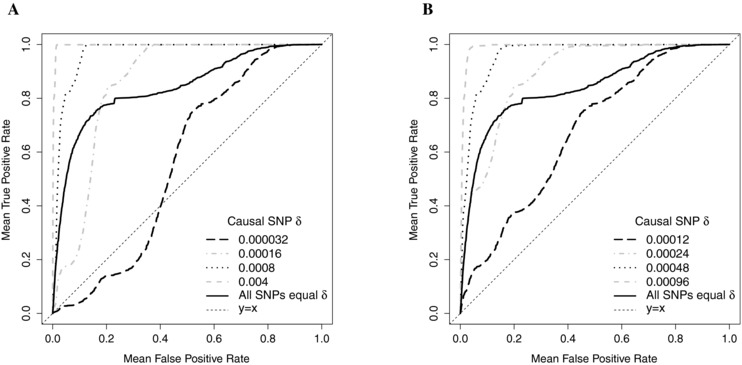
Effectiveness of posterior probability of causal association (Δ) as a fine‐mapping filter according to the prior probability of causal association (δ) of the causal SNP. One thousand datasets were simulated for each of four scenarios using causal SNPs with per‐allele OR of 1.1, MAFs close to 0.04 and a total sample size of 20,000 using the LD structure of the *CASP8* region. All SNPs were assigned to one of four prior probability groups and for each scenario a different causal SNP was selected so that it came from each of these four groups. A prior on the logOR of $N(0,W_{\mathrm{EB}})$ and the Wakefield approximation were used with $W_{\mathrm{EB}}={\hat{\beta_{1}}}_{p=30}^{2}-V_{m}$. (a) ROC curves for each of the four prior probability scenarios when the values of δ assigned to the SNPs in the four groups were 0.000032, 0.00016, 0.0008 and 0.004 (R=5). An ROC curve of the results for filtering using BF alone is given for comparison. (b) ROC curves for each of the four prior probability scenarios when the values of δ assigned to the SNPs in the four groups were 0.00012, 0.00024, 0.00048 and 0.00096 (R=2). A ROC curve of the results for filtering using BF alone is given for comparison.

Using these prior probabilities, we calculated the posterior probabilities (Δ) by updating with the BF using $W_{\mathrm{EB}}={\hat{\beta_{1}}}_{p=30}^{2}-V_{m}$. Figure 4a shows the results of filtering for the simulated data using Δs when the causal SNP is placed in each of the four SNP groups with different prior probabilities. The results of filtering using BF alone for one of the four scenarios has also been included for comparison. The BF results were very similar for all four scenarios with area under the curves (AUCs) of approximately 84%. As expected, when the causal SNP was in group 1 with a low δ value, it was a lot less likely to be retained than when it was assigned any of the other possible δ values. The AUC of the ROC curve for this scenario is 56%. Causal SNPs with the other three δ values all resulted in higher TPRs than BF filtering at FPRs greater than 0.18 and the AUCs for these scenarios are 86%, 97% and greater than 99% for groups 2, 3 and 4, respectively.

If the causal SNP is in group 3 or 4, the TPR is greater than 0.95 at FPRs as small as 0.11. When the values of $\delta_{\left[j\right]}$ are assigned using this method, the $\delta_{\left[j\right]}$ values depend upon the relative numbers of SNPs in each group as can be seen in Equation (5).

An *R* value of 5 is high, reflecting the expert's understanding of the disease mechanism. If less is known about the disease, investigators may specify lower values. To assess the effect of a lower value of *R* we did the same analysis with R=2. The results are presented in Figure 4b. The ROC curves for filtering using Δ are all closer to the BF ROC curve, particularly for groups 1 and 2. When the causal SNP is in group 2, 3 or 4, the ROC curves in Figure 4b have larger AUCs (89%, 96% and 99%) than the ROC curve for filtering using BF alone (AUC = 84%). However, the AUC is quite a lot smaller when the causal SNP is in group 1 (67%).

We also compared the results of posterior probability filtering with R=5 to those using R=2 and BF filtering by examining the numbers of SNPs (both causal and non‐causal) retained when the TPR is fixed at 90% when the causal SNP is each of the four groups. These results are given in Table 4. The results show that it is possible to reduce the set of candidate causal SNPs to 28 of 2,871 with posterior probability filtering at a TPR of 90% using available functional information. These results also indicate that if SNPs cannot be confidently assigned to prior groups based on functional information, then BF should be used for filtering (equivalent to assigning all SNPs the same value of δ).

**Table 4:** The numbers of SNPs retained out of the total 2,871 in the region, such that the true‐positive rate (TPR) is 0.9

		R=5		R=2	
Causal SNP group	BF filtering:	Δ filtering:	Inter	Δ filtering	Inter
(prior	mean (SD)	mean (SD)	section:	mean (SD)	section:
probability)	*BF threshold*	Δ *threshold*	mean (SD)	Δ *threshold*	mean (SD)
Group 1	1,514 (351)	2,059 (211)	1,514 (351)	1,926 (284)	1,514 (351)
(δ_[1]_)	*0.84*	*2.7* × *10* ^−5^		*1.0* × *10* ^−4^	
Group 2	1,515 (433)	814 (124)	638 (182)	823 (156)	692 (184)
(δ_[2]_)	*0.88*	*1.4* × *10* ^−4^		*2.1* × *10* ^−4^	
Group 3	1,701 (457)	264 (61)	255 (65)	315 (76)	309 (78)
(δ_[3]_)	*0.83*	*6.6* × *10* ^−4^		*4.0* × *10* ^−4^	
Group 4	1,678 (441)	28 (12)	28 (12)	62 (42)	62 (42)
(δ_[4]_)	*0.83*	*3.3* × *10* ^−3^		*8.0* × *10* ^−3^	

For four scenarios with similar causal SNPs (each in a different prior probability ($\delta_{\left[j\right]}$) group), Bayes factor (BF) filtering was carried out and the results are given in the second column. Posterior probability (Δ) filtering was carried out with group‐specific $\delta_{\left[j\right]}$ values assigned in two different ways, as indicated in the top row. Results are given as the mean and standard deviation (SD) of the numbers of SNPs retained and the BF or Δ threshold required to achieve this TPR. We also provide the mean and SD results for the intersection of SNPs retained using BF and Δ filtering. For each scenario, 1,000 datasets, with a causal SNP with a per‐allele OR of 1.1, an MAF in the range (0.037, 0.049) and a sample size of 20,000 was simulated using the LD structure of the *CASP8* region. To calculate the BFs, a prior on the logOR of $N(0,W_{\mathrm{EB}})$ and the Wakefield approximation were used with $W_{\mathrm{EB}}={\hat{\beta_{1}}}_{p=30}^{2}-V_{m}$.

### SNP Filtering in the iCOGS Data Using Posterior Probabilities

We fitted univariate logistic regression models to the iCOGS *CASP8* data from which we were able to calculate $W_{\mathrm{EB}}={\hat{\beta_{1}}}_{p=30}^{2}-V_{m}\approx 0.0018$. We used this value of *W* to calculate an approximate BF for each SNP. Due to the set of *CASP8* SNPs in the study being different to those in the 1,000 genomes data used for simulations, the number of SNPs assigned to each prior probability group was different. The numbers are provided at the bottom of Figure 3. Using Equation (5) with $p_{0}=0.4$ and both R=5 and R=2 gave the prior probabilities of association in Table 5.

**Table 5:** SNP group prior probabilities used in the iCOGS analysis for R=5 and R=2

	Prior probabilities of association			
Value of *R*	δ_[1]_	δ_[2]_	δ_[3]_	δ_[4]_
R=5	$5.44\times 10^{-5}$	$2.72\times 10^{-4}$	$1.36\times 10^{-3}$	$6.8\times 10^{-3}$
R=2	$2\times 10^{-4}$	$4\times 10^{-4}$	$8\times 10^{-4}$	$1.6\times 10^{-3}$

In Table 6, we present the top 20 SNPs in the iCOGS *CASP8* data ranked by posterior probability of causal association (Δ) using R=5. For notational purposes we use $\Delta^{\left[R\right]}$ to indicate the value of *R* used to calculate Δ. Table 6 also contains the rankings using Δ^[2]^, BFs and the more standard *P*‐values for comparison. These sets of results demonstrate the difference that including prior information can make. Although BF ranking is based purely on the data from the study and sets equal prior probabilities of causal association for each SNP, using Δ^[5]^ specifies the priors to be markedly different across the SNP prior groups. When using Δ^[2]^, there is less prior difference across the SNP groups, thus each SNP is ranked somewhere between the rank using Δ^[5]^ and the rank using the BF. Because of the large sample size, the likelihood is highly informative even when using Δ^[5]^. This is evidenced by the fact that the top 16 SNPs ranked by BF alone are all still in top 20 ranked by Δ^[5]^, so that the prior probability does not dominate the likelihood. However, we also see that in the top 20 ranked by Δ^[5]^, there are no group 1 SNPs (which are assigned the smallest prior probability). In fact the highest ranked group 1 SNP is ranked 87th using Δ^[5]^, but has a BF of 101 that is higher than some of the group 3 and four SNPs in the table (it is ranked 28th by BF and 39th by Δ^[2]^). A previous study [Spencer et al., 2015] quantified the variation in ranks using BFs as a function of sample size and hence implicitly the relative influence of the priors.

**Table 6:** Top ranked SNPs in *CASP8* region based on posterior probability (Δ) filtering using R=5 in the iCOGs study (89,050 subjects and 1,733 SNPs)

						Δ filtering				
				BF filtering		R=2		R=5		
SNP number	OR (95% CI)	MAF	*P*‐value filtering rank	BF	Rank	Δ	Rank	Δ	Rank	Group
838	1.041 (1.020, 1.062)	0.338	5	330	5	0.346	3	0.693	1	4
837	1.042 (1.021, 1.064)	0.299	6	306	10	0.329	4	0.677	2	4
1,027	1.046 (1.024, 1.068)	0.285	2	955	2	0.433	2	0.565	3	3
980 b	1.048 (1.027, 1.071)	0.294	1	1,932	1	0.436	1	0.345	4	2
896	1.042 (1.020, 1.064)	0.287	13	254	=12	0.169	5	0.257	5	3
885	1.041 (1.019, 1.063)	0.287	19	184	16	0.129	7	0.201	6	3
893 a ^,^ b	1.080 (1.032, 1.131)	0.047	42	30	45	0.046	25	0.170	7	4
839 b	1.035 (1.013, 1.057)	0.270	54	26	=50	0.040	29	0.151	8	4
992 b	1.045 (1.022, 1.067)	0.287	3	488	3	0.163	6	0.117	9	2
909	1.043 (1.021, 1.065)	0.287	9	352	4	0.124	8	0.087	10	2
950 b	1.043 (1.021, 1.065)	0.286	10	326	6	0.115	9	0.081	11	2
960 b	1.043 (1.021, 1.065)	0.285	7	320	=7	0.114	=10	0.080	=12	2
961 b	1.043 (1.021, 1.065)	0.285	8	320	=7	0.114	=10	0.080	=12	2
985 b	1.043 (1.021, 1.066)	0.286	4	310	9	0.111	12	0.078	14	2
907	1.042 (1.020, 1.064)	0.287	6	255	11	0.093	13	0.065	15	2
912	1.042 (1.020, 1.064)	0.287	15	254	=12	0.092	14	0.065	16	2
890	1.028 (1.008, 1.048)	0.386	141	9	149	0.014	45	0.057	17	4
956 a ^,^ b	1.052 (1.025, 1.080)	0.167	16	210	14	0.078	15	0.054	18	2
1,272 a	1.075 (1.036, 1.116)	0.071	14	190	15	0.071	16	0.049	19	2
971 a ^,^ b	1.083 (1.034, 1.135)	0.049	31	40	34	0.028	30	0.048	20	3

^a^For these SNPs, the major allele is associated with a higher disease risk.

^b^These SNPs were not genotyped but imputed.

Also included for comparison are the ranks of these 20 SNPs when using posterior probabilities with R=2 and Bayes factors (BF) calculated using the Wakefield approximation with $W_{\mathrm{EB}}={\hat{\beta_{1}}}_{p=30}^{2}-V_{m}=0.0018$, as well as *P*‐values. The estimated OR (with 95% confidence interval) and MAF for each SNP are also included.

Including prior information can help to pinpoint the causal signal in blocks of high LD that ranking by BF, or any statistic calculated from the genotype data alone, cannot. Many of the SNPs in Table 6 are in very high LD with each other. For example, 12 of the SNPs in the top 13 ranked by BF all have an estimated OR in the range (1.042, 1.048) and a sample MAF in the range (0.285, 0.299). Ranking by Δ^[5]^ not only changes the ranks for these 12 SNPs within the LD block, increasing the weight given to those SNPs with higher prior probabilities (e.g., SNP number 837), but also includes in the higher ranks SNPs from outside the LD block. For example, SNP number 893 (with an estimated OR of 1.080 (95% CI=(1.032, 1.131)) and MAF of 0.047) is ranked 45th by BF but is ranked seventh when ranked by Δ^[5]^.

The 173 top ranking SNPs (top 10%) using Δ^[5]^ contain only 93 in the top 10% ranked by BF. Of these 173 SNPs, 15 were assigned the highest prior probability, 92 the next highest and 62 and 4 the two lowest probabilities, respectively. These are equivalent to 100%, 41%, 12% and 0.4% of the total SNPs assigned to groups 4, 3, 2 and 1, respectively. The SNP in the top 10% based on Δ^[5]^ that is ranked lowest by BF is SNP number 889, which only has the 1,731st highest BF value (out of 1,733), but due to having been assigned the highest prior probability, it is ranked 169th by posterior probability.

## Discussion

BFs provide a coherent framework for combining information from a genetic association analysis with information from other sources. We have developed an empirical Bayesian prior distribution for the logOR (β_1_) to use with the Wakefield BF approximation and also propose a general framework for assigning prior probabilities of association (δ) to genetic variants, combining functional SNP data with expert elicitation. Through simulation, we showed that when using the WBF approximation with a prior of the form $\beta_{1}∼N\left(0,W\right)$ to filter SNPs in the *CASP8* region, using $W=W_{\mathrm{EB}}={\hat{\beta_{1}}}_{p=30}^{2}-V_{m}$ generally gave higher TPRs for a given FPR across a range of values of *W* likely to be used in fine‐mapping studies looking for variants with modest effect sizes. We found that using the median of the absolute value of the effect size estimates in the top 30% (p=30) ranked by likelihood performed well in our simulated data but our investigations were limited to the *CASP8* region and a different value may be needed in a different region with substantially different LD structure.

The method will be most effective in fine mapping a single causal variant. Multiple causal variants with different MAFs could possibly lead to different effect size estimates (under the assumption that rarer variants have larger effect sizes). Indeed even two causal SNPs with similar effect sizes could also have disparate effect size estimates in small sample sizes. In regions harbouring a single causal variant this is obviously not an issue. So the effectiveness does not necessarily depend on variant MAF, but on the presence of a single causal SNP. The method may work well in regions with multiple causal SNPs, if the causal SNPs have similar effect sizes and the sample size is large, because then the estimates are likely to be similar for SNPs in high LD with either causal SNP. Care should be taken in smaller fine‐mapping studies because rare variants may have relatively high effect estimates. Inclusion of these rarer variants in the top *p*% of variants may lead to unreliable estimates of the effect size of the causal SNP although taking the median over these effect sizes should mitigate this somewhat.

This work was motivated by the large quantities of SNP‐level functional data now freely available online. Incorporating external data such as these could be a way of disentangling the signals coming from high LD regions harbouring a causal SNP, a problem invariably encountered in fine‐mapping studies. A common methodology to differentiate between the large number of SNPs in a region is to use the results of genome‐wide association study (GWAS) and then systematically examine functionality databases to justify the top hits. We have formalised the incorporation of functional information by using it to inform the prior probability of causal association.

### Incomplete Functional Data

The functional encode SNP‐level data that we used to assign specific prior probabilities for each SNP group currently have the disadvantage of not being complete for all the SNPs across the genome and in fact is quite sparse for some functional indicators. This means that in addition to potential uncertainty around the prior probabilities, there is likely to also be uncertainty about how to handle SNPs for which there is some functional information missing. We dealt with missing values in the encode data by replacing them with zeros under the assumption that they are missing because they didn't exceed some measurement threshold, but it is unclear how appropriate this is. It may be more robust to impute these missing values, using the recorded values for other SNPs showing reasonable correlation in some set of ENCODE variables. This approach would be particularly challenging because the functional effect of an SNP depends on the sequence around it and so this would have to be taken into account in the imputation in some way. Understanding the reasons for the data being missing provides the key to knowing how best to handle them.

### Alternative Methods of Including Functional Data

Although data external to the association study are not often used in the initial analysis, there are several structured methods other than through BFs in which it can be in incorporated. These include *P*‐value weighting [Saccone et al., 2008] a Bayesian latent variable model [Fridley et al., 2011] and stratified false discovery rates [Schork et al., 2013; Sun et al., 2006].

There are also different methods by which δ values could be assigned to variants in a study. An alternative method of grouping is to obtain SNP scores from the RegulomeDB database [Boyle et al., 2012]. These categorical scores are assigned based on the regulatory potential of variants and draw information from multiple sources including encode [Encode Project Consortium, 2011]. In this case, the score is between 1 (for most likely to be causal) and 7 (for least likely). Rather than grouping, a different strategy is to use some sort of continuous score for SNPs. Several such scoring methods have been published recently, based on an SNP's individual probability of affecting disease susceptibility, for example, the functional significance (FS) score published by Lee and Shatkay [2009], which has been used effectively to enhance expression quantitative trait loci (eQTL) fine mapping [Boggis et al., 2015]. The FS score has the advantage that it integrates a large amount of data from multiple publicly available data sources. It formally combines scores from a number of bioinformatics tools using weighting based on the reliability of these tools to give a score between 0 and 1.

An alternative way to integrate functional information into this kind of analysis is to use it to form a BF rather than a prior probability [Knight et al., 2011]. This method is effective because ‘prior knowledge’ can be updated any number of times using BFs. Once a posterior odds of association has been calculated, this can be used as a prior odds and multiplied by another BF to get a new posterior odds. Therefore, beginning initially with all SNPs having equal prior probabilities of association, two separate BFs can be used, one containing the association information from the genotyping, as detailed in this study, and the other containing the functional information. Knight et al. [2011] give some specific values that may be used for these functional BFs.

Pickrell [2014] jointly analyses functional genomic data and GWAS data. The approach taken is an objective, rather than a subjective one. In a block of SNPs assumed to harbour a single causal variant, the prior probability that an SNP is causal depends on annotation parameters that are jointly estimated along with all other parameters. This is an interesting approach but because our interest was in breast cancer, a disease with a relatively well understood aetiology, we felt that it was appropriate to use the accumulated subjective knowledge of a breast cancer expert. This may not be the case in less well‐understood diseases.

Functionally informative data are expected to become more complete in the near future and large databases such as encode are regularly updated. We anticipate that methods such as those described here will become increasing important as these data become more complete.

## Justification for Maximising WBF with Respect to *W* in the Empirical Bayes Approach

Here we justify our assertion that f( data |W)∝ WBF  when considered as a function of *W*. The WBF is given by $f\left(\mathrm{data}|H_{1}\right)/f\left(\mathrm{data}|H_{0}\right)$ and because $f(\mathrm{data}|H_{0})$ imposes the constraint that β=0 it does not involve *W*. It follows that WBF $\propto f(\mathrm{data}|H_{1})$ when considered as a function of *W*. Under *H*_1_ the support for β is β∈(−∞,0)∪(0,∞), whilst the support for β in the prior used in the marginal likelihood f( data |W) is β∈R. However the Lebesgue measure ensures that P(β=0)=0 and so $f\left(\mathrm{data}|H_{1}\right)=f\left(\mathrm{data}|W\right)$ even though the first involves integration over β∈(−∞,0)∪(0,∞) whilst the latter involves integration over β∈R. It follows therefore that f( data |W)∝ WBF  when considered as a function of *W*.

## Conditions, Derivation and Classification of Stationary Point

Let K=V exp (β1^2/2V), which is not a function of *W*. Because *W* represents the variance of a (possibly degenerate) random variable, it follows that W≥0. Let f(W) represent the WBF in Equation (2) as a function of *W* then

f(W)=VV+W exp β1^2W2V(V+W)=VV+W exp β1^22V1−VV+W=K(V+W)−1/2 exp −β1^22(V+W).

After a little algebra it follows that

f′(W)=K(V+W)−3/22β1^2−(V+W)V+W exp −β1^22(V+W).

So $f^{\prime }\left(W\right)=0$ when $W={\hat{\beta_{1}}}^{2}-V$. If $W={\hat{\beta_{1}}}^{2}-V\geq 0$ then $W={\hat{\beta_{1}}}^{2}-V$ is a stationary point. If ${\hat{\beta_{1}}}^{2}-V<0$, then ${\hat{\beta_{1}}}^{2}-\left(V+W\right)<0$, the derivative is strictly decreasing and W=0 is a (non‐stationary) maximum. A little more algebra gives

f′′(W)=K4(V+W)−5/23−6β1^2V+W+β1^4(V+W)2 exp −β1^22(V+W)f′′(W=β1^2−V)=−K2(β1^2)−5/2 exp −1/2.

So if ${\hat{\beta_{1}}}^{2}-V\geq 0$ then the stationary point $W={\hat{\beta_{1}}}^{2}-V$ is a maximum. If ${\hat{\beta_{1}}}^{2}-V<0$ then f(W) is maximised at W=0. If follows that f(W), the WBF, is maximized at

WEB=β1^2−V if β1^2≥V0 otherwise .
